# Digital Twin Generation: Re-Conceptualizing Agent Systems for Behavior-Centered Cyber-Physical System Development

**DOI:** 10.3390/s21041096

**Published:** 2021-02-05

**Authors:** Christian Stary

**Affiliations:** Business Informatics-Communications Engineering, Johannes Kepler University Linz, 4040 Linz, Austria; christian.stary@jku.at; Tel.: +43-732-2468-4320

**Keywords:** digital twin, behavior modeling, Cyber-Physical Systems, design-integrated engineering, subject orientation, intelligent agents

## Abstract

Cyber-Physical Systems (CPS) form the new backbone of digital ecosystems. Upcoming CPS will be operated on a unifying basis, the Internet of Behaviors (IoB). It features autonomous while federated CPS architectures and requires corresponding behavior modeling for design and control. CPS design and control involves stakeholders in different roles with different expertise accessing behavior models, termed Digital twins. They mirror the physical CPS part and integrate it with the digital part. Representing role-specific behaviors and provided with automated execution capabilities Digital twins facilitate dynamic adaptation and (re-)configuration. This paper proposes to conceptualize agent-based design for behavior-based Digital twins through subject-oriented models. These models can be executed and, thus, increase the transparency at design and runtime. Patterns recognizing environmental factors and operation details facilitate the configuration of CPS. Subject-oriented runtime support enables dynamic adaptation and the federated use of CPS components.

## 1. Introduction

Cyber-Physical System (CPS) are dynamic and complex systems, as their design involves a variety of disciplines, leading to contributions by various teams of engineers and experts with diverse backgrounds [1]. The latest digital infrastructure strategies, such as the one developed by Gartner (https://www.zdnet.com/article/gartner-sees-internet-of-behaviors-automation-ai-experiences-key-2021-technologies/#:~:text=Internet%20of%20Behaviors%2C%20or%20IoB,IoB%20commercial%20or%20government%20program) (accessed on 4 February 2021), suggest a unifying behavioral approach to operation, the Internet-of-Behaviors (IoB), as a follow-up to the Internet-of-Things (which can be considered an inherent part of most CPS). IoB delivers operations, automation, and total user experiences anywhere. CPS technologies thereby require “plasticity” or the ability to adapt to changing conditions; the future design of CPS requires not only behavior specifications but becomes a moving target and needs to be coupled with continuous engineering. It requires technical and technological capabilities where almost every second professional workforce should be able by 2023 to orchestrate business applications in a self-managed way (https://www.gartner.com/smarterwithgartner/gartner-top-strategic-predictions-for-2020-and-beyond/) (accessed on 4 February 2021). It will become part of the total user experience mentioned above. CPS development itself is then highly heterogeneous, as it involves customers, employee, and design experiences considered to be critical amid distributed organizations. It is becoming a socio-technical endeavor.

The diversity of expertise and active stakeholder roles in the design and engineering of CPS influences system design and operation and requires high-level integrated design methods, according to a survey study [2]. Therefore, system modeling forms the foundation for the structure and operation of developed CPSs. The CPS design process requires focus on the (1) physical components, (2) digital components, and (3) their integration, preferably in digital representations, termed digital twins. The physical components are those CPS parts that are present in the physical world. According to their capabilities, they recognize events and can set actions physically, mainly through sensors and effectors/actuators, respectively. Digital components represent all other processing-relevant CPS parts. Design is finally about composing and aligning these components in a way that the required CPS functionality can be accomplished through their interplay in socio-technical settings, thus qualifying CPS as socio-technical systems.

Traditionally, CPS designers directly start working on functional and architectural issues rather than developing a conceptual model (i.e., digital twin representations) [2]. Hehenberger et al. [2] also concluded from existing works that languages for modeling need to refer to architecture issues of CPS. Such relations must also be aware of the specific nature of CPS. In that respect, the modular structure and dynamic design of CPS are of crucial importance. Hence, component interface design requires particular care, since, on the one hand, CPS parts need to operate in a federated way, and, on the other hand, functionality should adjust to the optimal use of existing resources.

Khaitan et al. [3] referred to CPS as a “system of systems” due to their federated architecture, despite their heterogeneity and complexity. Such a system understanding should help throughout the design process to model domain specific, continuous interaction and adaptation according to the changing configurations. The reported list of modeling techniques and programming tools for designing CPSs include formal semantics approaches and architecture styles, such as (multi-)agent and event-based semantic models. These models should feature intelligent composable (business) operations (https://www.zdnet.com/article/gartner-sees-internet-of-behaviors-automation-ai-experiences-key-2021-technologies/#:~:text=Internet%20of%20Behaviors%2C%20or%20IoB,IoB%20commercial%20or%20government%20program) (accessed on 4 February 2021). Composable designs facilitate flexible and resilient architectures and composable technologies. These technologies lay ground to automating business and IT processes.

However, building up intelligence requires explainable representations, as recent projects in developing intelligent CPS show, e.g., https://uranos.ch (accessed on 4 February 2021): “… many current concepts of AI are overestimated, and their applications are insufficient for the everyday lives of people work. Further, we argue that holistic and human-centered systems will become more important so that machines and people can work together better. Our work focuses on the modeling and implementation of man-machine conversation and related learning between man and machine by sharing knowledge.” This call for human-centered CPS reveals the need of technology-based mechanisms for a new generation of support tools that can effectively guide engineering practices related to intelligent system development.

Consequently, this paper focuses on the behavior-centered design and control capturing the aforementioned heterogeneity. The diverse and dynamic nature of CPS requires introducing composable and executable digital twins as behavior models. They are part of development that integrates the operating user/consumer into design processes and puts modeling as the center of CPS (development) practice. Such an integrative approach comprises “exploration” as the divergent top-down design, “synthesis” as the selection for integration, and “development” as the convergent bottom-up design [4]. CPS design-integrated engineering becomes an active user role, in addition to domain-specific or technical user roles. CPS control activities comprise operating a CPS in a specific functional user role and, additionally, in a combined designer-engineer role further developing functional roles and, thus, the CPS.

**Figure 1:**
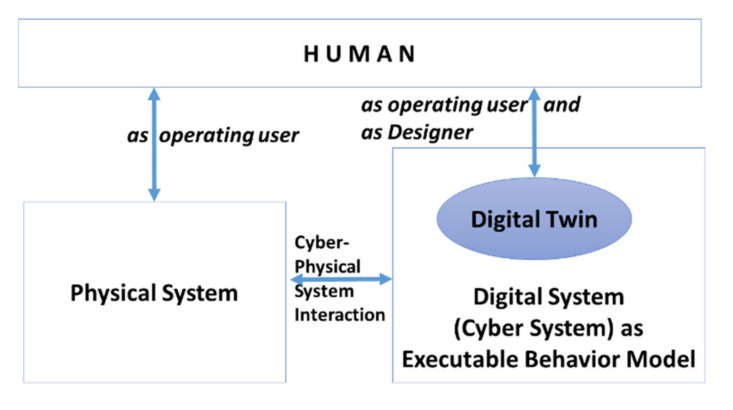
Cyber-Physical Systems as a socio-technical system with digital twin representation for functional operation and design-integrated engineering.

Figure 1 shows the architecture of CPS as a system, including its executable behavior model as a digital twin enabling design and control in terms of modeling, validation, and execution. In the following, we use the term design-integrated engineering for the design and control of digital twins in terms of modeling, validation, and execution. In that context, users are either an operating workforce or designers adapting the digital twin for further control. The benefit of mirroring a CPS through executable design models is not only the adaptability at runtime when CPS (component) behavior is modified or extended, driven by user needs, but, also, the switching of (physical) implementations for technological reasons, e.g., replacing activities through robotic movements or adding decision support for nonroutine business cases. Therefore, the executable behavior models need to represent the CPS in an implementation-independent form.

In the following section, I detail the methodological cornerstones of my research to achieve this objective. For the sake of effectiveness and accuracy, I primarily utilized the existing knowledge and experiences on the design-integrated engineering of behavior models (digital twins) for CPS development. They stem from an agent-based system and business process development.

## 2. Methodology

In this section I argue how CPS developers can learn from agent-based systems and demonstrate common grounds with CPS before I explain methodologically the mapping of CPS components to model entities for design-integrated engineering. In order to demonstrate the results, I argue for including a use case—namely, transforming a grocery shop to a CPS. It is used throughout this study. Thereby, the methodological point of reference for CPS design and control are digital twins. Based on subject-oriented modeling, they enable representing and dynamically adaptable encapsulated behavior in both an easy-to-share and executable way. Finally, I motivate the stepwise presentation of the results that are given in the subsequent sections.

Conceptualizing Agents. Agent-based systems have become widespread in complex environments (cf. [5]). While the Oxford American Dictionary defines “agent” as “a person or thing that takes an active role or produces a specified effect” (http://oaadonline.oxfordlearnersdictionaries.com/dictionary/agent) (accessed on 4 February 2021), agents in socio-technical environments (e.g., business organizations) are active entities causing an environment to change (effects). They might act “on behalf of another, for example, managing business, financial, or contractual matters, or provide a service” (ibid.), such as intelligent recommending or rerouting. In line with this understanding, Sterling et al. [6] defined an agent as “an entity that performs a specific activity in an environment of which it is aware and that can respond to changes” (p. 7). Wooldridge [7] seconded that “an agent will have a repertoire of actions available to it. This set of possible actions represents the agent’s effectoric capability: its ability to modify its environment” (p. 6). Its actions have preconditions associated with them, and they capture “the possible situations in which they can be applied” (ibid.)—each attribute qualifies for CPS design.

Agent behavior has been termed “intelligent” when an action could be programmed deciding which of an agent’s actions is performed in order to best meet the demands of a situation or best satisfy delegated objectives to the agent. As such, agents are decision-making systems that are embedded in an environment. Like CPS, they are computer systems that are capable of autonomous action in some environments in order to meet objectives assigned to them by system designers—also termed goal-oriented behavior. A repertoire of actions can be executed “to modify the environment, which may appear to respond non-deterministically to the execution of these actions” (ibid.), either proactively or reactively, either as a stand-alone or in cooperation with other agents.

Hence, software agents representing CPS components can have the ability to perform tasks in an autonomous way. In that case, humans or other components do not intervene as part of the control flow or business logic. Even decision-making can be handled internally before an activity is set influencing the environment of the system. Since agents can operate independently from each other, as well as in collaboration, agent-based systems can be considered federated systems.

**Figure 2:**
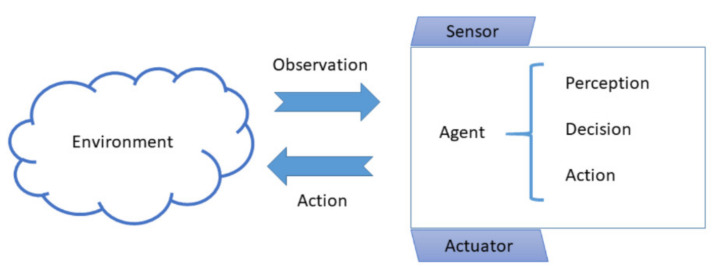
Conceptualizing Cyber-Physical System (CPS) components as agents encapsulating a behavior.

Figure 2 provides a conceptual view on agents that is used for CPS components modeling. As part of a living environment, agents are coupled with sensors to receive inputs from this environment and have actuators allowing them to create effects in their environment. The set of internal actions of encapsulated agent behavior is to analyze incoming information (“perception”), process it to identify valid behavior options (“decision”), and to set an action implementing the decision (“action”) via an effector or actuator.

**Figure 3:**
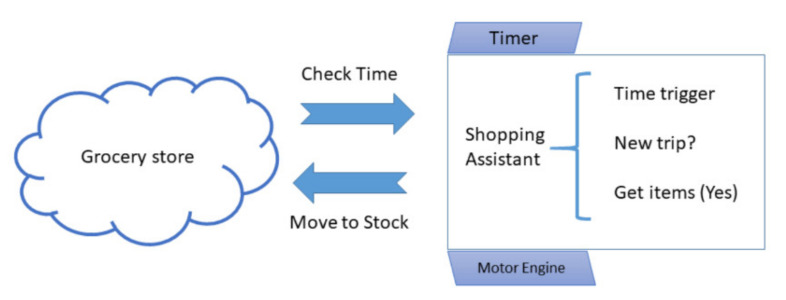
Simple grocery shopping assistant as an agent-based CPS component.

Transforming Grocery Store as Demonstrator. Figure 3 shows a traditional application of agent-based design utilizing the scheme from above. The shopping assistant component of a grocery store can be assumed an active CPS part. It could either be local to the user environment or remote in some location. It is a behavior abstraction serving the need of handling customer orders (cf. North et al. [8]). On one hand, it reacts on input signals (“Timer” in Figure 3); on the other hand, it actively influences other system components—for example, by being able to move around (“Motor Engine” in Figure 3). The behavior of the grocery shopping assistant is triggered by time signals (“Time trigger” in Figure 3). It behaves in the shopping environment according to the internal decisions taken. In the grocery use case, it checks whether a shopping assistant trip needs to be triggered (“New Trip?” in Figure 3). In case an item has to be delivered, it moves to stock to deliver it (“Get Items” in Figure 3).

In the following, I will use this setting to demonstrate behavior-driven CPS design and control, as many retail businesses transforming from traditional operation to digital ones enhance or extend their operations with physical components, such as drones or robotic systems. Such shifts become increasingly common in times of crises (See KPMG, 2020: https://home.kpmg/us/en/home/insights/2020/09/digital-acceleration.html and McKinsey https://www.mckinsey.com/business-functions/strategy-and-corporate-finance/our-insights/how-covid-19-has-pushed-companies-over-the-technology-tipping-point-and-transformed-business-forever) (accessed on 4 February 2021). The suggested behavior abstractions aim to avoid disruptive change processes through digital twins as digital threads. They mirror the behavior during design and runtime due to their implementation-independent modeling and execution capabilities. The blueprint for this type of digital twin stems from experiences with agent system development and sociotechnical, subject-oriented process development.

The fundamental grocery shopping assistant behavior is as follows: It waits for receiving orders or requests from customers. When browsing the “store” (which can be located in a single location or be arranged as distributed enterprise) handling the order, it checks target items. As a contextual component, it also checks hindrances and the related frustration of customers in the case of delays, physical distractions, and the mismatch of orders. In those cases, the assistant develops alternatives to complete an order and deliver the items according to the customer requests (cf. [8]).

Digital Twins Design. Agent behavior abstractions can be used for representing CPS through digital twins, i.e., allowing for behavior encapsulations that (i) can be executed automatically for operating, probing, and simulation and (ii) abstractly from physical and digital runtime evidence. However, a semantically precise diagrammatic representation scheme is required to ensure the correct execution of validated behavior. The suggested approach provides both and stems from Subject-oriented Business Process Management (S-BPM) (cf. [9]). It provides effective representations for CPS behavior abstraction, dynamic control, and adaptation. Digital twins as digital threads that can be executed through validated behavior models make CPS development more effective and efficient compared to traditional roundtrip engineering (cf. [10]). Digital twins benefit from developing behavior descriptions through component intelligence in dedicated representations—so-called Subject Behavior Diagrams. They are subsumed on the level of an overall CPS architecture scheme in so-called Subject Interaction Diagrams (see the following section).

Dynamic Control for Building up Intelligence. Once CPS as dynamically evolving systems through adaptation are handled by subject-oriented digital twins, CPS can be designed by users in operations interactively in a dual role as designer. They become organizational artefacts. Due to direct runtime support during design time, CPS can be developed along a design-integrated engineering process. Building up intelligence in this way the facilitates digital reference model generation of CPS represented as digital twins, as well as the standardization of designs. The simple notation of the subject-oriented representation scheme facilitates the emergence of behavior patterns that can serve as blueprint for further developments, refining and semantically enriching the fundamental agent pattern “perception—decision—action”. Reference models and patterns impact the implementation of the Internet of Behaviors (IoB) as the infrastructure for complex system semantically defined components that are arranged in a choreographic way to ensure autonomy and dynamic adaptation.

After having introduced the methodological cornerstones in this section, the following sections detail the inputs from digital twin research, agent-based systems, and subject-oriented behavior modeling. The addressed integration targets digital twin development: Section 3 contains essential background knowledge on the current digital twin specifications, agent-based systems, and subject orientation. It also details common grounds and design elements for the (re-)conceptualization of agent systems through subject-oriented design and execution capabilities. Section 4 explains the fundamental development steps of CPSs as socio-technical systems based on behavior models. Section 5 describes how the adaptive behavior of CPS components builds up intelligence based on subject-oriented modeling (patterns). Section 6 discusses the results and concludes the paper.

## 3. Digital Twins as Behavioral Design Encapsulations

Digital twin developments currently peak the Gartner Tech hype curve (see, also, https://www.gartner.com/smarterwithgartner/5-trends-drive-the-gartner-hype-cycle-for-emerging-technologies-2020) (accessed on 4 February 2021). The digital twin market evolves from machines and equipment health monitoring applications with about 34% compound annual growth rate through 2026 and is propelled by an increasing demand for real-time monitoring and equipment analysis through the IoT, creating research potential on verticals. CPS insights through digital twins are required that could otherwise be difficult, sophisticated, or simply impossible to discover (cf. https://www.newswire.com/news/digital-twin-market-revenue-to-hit-usd-35-bn-by-2026-global-market-21211854) (accessed on 4 February 2021).

I followed the recently consolidated conceptualization of digital twins proposed by Wagner et al. [11]. It reflects the nature of CPS as a digital twin that consists of respective parts physical and digital product and “connected data that tie and indissolubly connect the physical and virtual product”. It is considered an integrated simulation for optimizing a CPS while mirroring the lifecycle of its actual system pendant. Mirroring means to fully capture the potential or actual physical manufactured product from its micro or atomic level to the macro level through models. Beyond referring to the product a digital twin can contain a production system itself.

However, so far, digital twins are mainly used in the product design stages to gain quantitative data for efficient and optimal product generation along the value chain for product life cycles. Digital twins increasingly contain digital production information, so that production systems can be simulated. Besides, manufacturing steps (machine) tools and process parameters are handled in the course of optimization. Linking product and production twins into digital production twins affects the product engineering process, even addressing human control and interaction issues. Wagner et al. [11] envision enriched use of digital twins along product engineering to ensure the coherent and efficient flow of information. They also recognize the lack of a consistent framework for holistic application scenarios, particularly addressing the integration of product designers’ work and production planner concepts.

The latter has already been addressed recently by Papacharalampopoulos et al. [12]. They proposed to study what-if scenarios through experimenting with varying process parameters. They are handled by real-time digital twins, thus requiring executable models representing relevant design parameters. Therefore, the authors recognized the requirement for validation, conceding that developments for manufacturing operation need to undergo thorough model checking in terms of semantic behavior qualities. The particularity of CPS to the author’s conceptual understanding concerns the capability of a digital twin representation to be able to make decisions in real time. In this respect, the digital twin differs as a model from physical representations of models of a product. In the course of optimization, human interventions when experimenting with different designs become transparent and traceable.

Consequently, a digital twin becomes a virtual instance bound to a physical system that is not only continually updated with the latter’s operation data on performance and status throughout the entire physical system’s life cycle but could control the overall system’s behavior during operation and development. In addition, the models representing the digital twin can address CPSs from different perspectives and in different layers based on networked micro and macro elements (cf. [11,13,14]).

In order to learn from technology developments concerned with dynamic system behavior, as described above, I encourage conceptualizing an established engineering approach to that respect. Agent systems provide the fundamental capability of flexible autonomous action in dynamic, unpredictable, and open environments while keeping the benefits of modular, component-based system design. In particular, for semantic computing, agent-like models can support the automation and semi-automation of information identification, collection, and processing tasks, in cooperation with other components. Typical applications are (product) recommender systems based on customer preference models (cf. [15]). However, agent-like models could play a manifold role in CPS engineering. Agent systems

- represent a metaphor when designing complex, distributed intelligent systems in terms of autonomous units of action.
- provide a set of technologies for orchestrating intelligence.
- allow the homo-morphological modeling of real-world scenarios and, thus, enable meeting human-centered needs in digital systems, e.g., with respect to organizing work.

For CPS design, we consider CPS components as kinds of agents or behavior entities that should be able to respond dynamically to changing circumstances in a self-contained way while contributing to a commonly agreed-upon goal. Such an overarching objective determines the degree of autonomy for each component, as CPS development requires the aggregation of different functionalities that might have previously been distinct (such as planning, doing, and coordinating). It needs to be achieved in a conceptually embodied and situated way.

According to the concept of digital twins, digital representations play a substantial role. Technologies needs to relate directly to abstractions in the design and execution of systems, as CPS components interact to achieve a common goal. Hence, the considerations at the macro level include issues of behavior decomposition, information architecting, communication, and workflow. The current agent technologies meet these objectives in terms of distributed planning and decision-making, bidding, communication, coordination, and negotiation, as well as learning (cf. [7]). As such, they can serve as conceptual role models for a variety of CPS development and operation tasks.

Business engineering and the servitization of production, which represent the context of CPS development in organizational settings, are mainly driven by the functional decomposition of value chains subsuming operational processes (cf. [16]). Subject-oriented BPM takes a behavior-centered perspective on these systems and processes, aiming for standardized, simple protocols and encapsulation, as advised by practitioners (Cf. https://www.heflo.com/blog/bpm/business-process-design-principles/) (accessed on 4 February 2021) and discussed above:

- Behavior encapsulations as a part of process representations have two constitutive elements: active entities (termed subjects) and their mutual interactions. Both are represented in Subject Interaction Diagrams (SIDs). Each of those active entities encapsulate behavior sequences, explicitly representing both functional activities (e.g., task performance) and communication—namely, sending and receiving messages. Encapsulated behavior is represented in Subject Behavior Diagrams (SBDs). Consequently, each subject of an SID is detailed in terms of encapsulated behavior through an SBD.
- Subjects’ interactions include data. Therefore, they exchange messages. These messages can include data, e.g., (business) objects, which are processed by the receiving subject (as detailed in the SBD of the receiving subject).
- Subject representation abstracts from organizational implementation. Since subjects are behavior abstractions, by the time of modeling, e.g., when designing a CPS (component), it is not specified whether a subject’s behavior is instantiated by humans, digital or physical systems, or a combination of those. This property is essential for representing digital twins as a baseline and for control purposes (see above). For instance, the grocery shop can either be a web shop or a physical one or any combination that can be controlled by a subject-oriented digital twin. The activities of shopping assistant can either be performed by humans, digital entities, physical systems, or a combination of those.
- Subject representations are executable. The control flow of each subject specifications contains functional and interaction activities that can be executed automatically for validation and operation. Like in reality, the runtime behavior of a subject-oriented model is designed to operate subjects in parallel. They exchange messages in an asynchronous or synchronous way, leading to a concurrent distributed system. For the automated execution of subject-oriented models, several tools have been developed, including Metasonic (see metasonic.de), actnconnect (see actnconnect.de), and UeberFlow (see i2pm.net). The CPS behavior in terms of business operation can be validated and experienced interactively before putting it into operation.
- Subjects act autonomously in a network. Any system or process consists of autonomously acting components, forming a network of interacting entities (subjects) to achieve a common goal. Besides sending and receiving messages, subject actions include local actions that do not involve interactions, such as calculating a value, and interactions, i.e., message exchanges.

**Figure 4:**
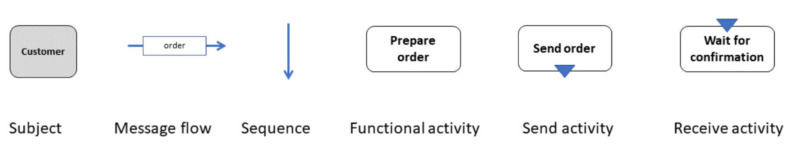
Diagrammatic Subject-oriented Business Process Management (S-BPM) elements for modeling.

Subject-oriented models, SIDs and SBDs, have five elements, as shown in Figure 4. Subject Interaction Diagrams (SIDs) operate with two symbols, whereas Subject Behavior Diagrams (SBDs) use three symbols. The five symbols are:

(i) A subject is identified by a name and represents a role in a process.
(ii) A message has a label and represents the exchange of information between subjects.
(iii) A functional activity or action is identified by a functional state name and represents a local action to be executed.
(iv) A send activity corresponds to a named send state and represents a communication action concerned with sending a message.
(v) A receive activity corresponds to a named receive state and represents a communication action concerned with receiving a message.

**Figure 5:**
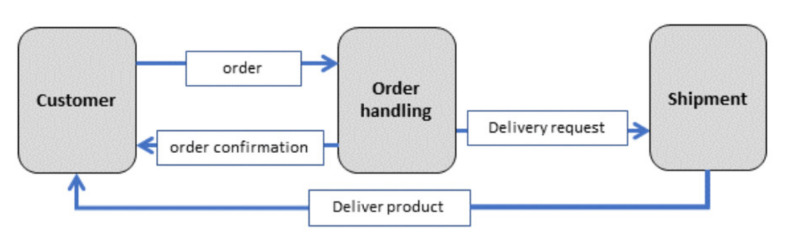
Subject Interaction Diagram for Order Handling, including subjects and messages.

**Figure 6:**
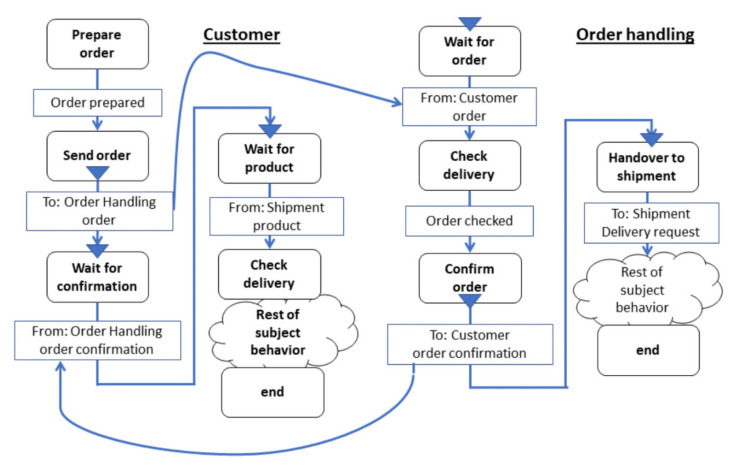
Order Handling—Subject Behavior Diagrams “Customer” and “Order Handling”.

For the global view on a process in a Subject Interaction Diagram (SID), a symbol is used for representing the subjects involved, and another symbol is used for the messages exchange between the subjects. Figure 5 depicts a SID of an ordering process. Figure 6 provides a sample Subject Behavior Diagrams (SBDs) detailing the business logic from the perspective of individual subjects abstracted from the actual implementation by CPS components. Each functional and communication element of an SBD corresponds to a state representing a local action or communication action for sending messages and receiving messages. The transitions between states are encoded by arrows in SBDs, whereby labels (displayed in rectangles) indicate the outcome of the preceding state or interaction information to a receiver or from a sender. The representation of functional states and message passing as control flow ensures automated model execution and, thus, the interactive probing of CPS (components) before being put to operation.

Since subject-oriented representations are abstract from implementation issues in the course of modeling, agent system specifications can be conceptualized by subject-oriented behavior encapsulations on the basis of communication protocols when using SIDs and behavior specifications enabled by SBDs. However, particularities such as semi-structured communication languages and design methodologies [17], need to be taken into account. In particular, ad-hoc, nondeterministic, and domain-specific requirements demand specific attention (cf. [18] for healthcare). The ultimate stage of scalability could be reached through the dynamic and intelligent formation of components and system architectures beyond domains, referring to generic adaptation capabilities (cf. [6]). In accordance with the agent-systems’ approach to distributing intelligence, (re-)conceptualization can build on the basic properties of intelligent agents as autonomous and proactive entities with social capabilities, as well as on the features of multiagent systems. These are the “management of distributed information, communication and coordination between separate autonomous entities” ([18], p. 9).

When business operation forms the context of agent systems, transparent specification and processing efficiency have been brought into play (cf. [6], p. 6): “There is a need and expectation for instantaneous responses, which will be achieved only by efficient implementations in light of the complexity. Efficiency may well determine the possibility of solutions—for example, file sharing or downloading large video files. A more abstract attribute is purposefulness. In light of the complexity and changing nature of the environment, it will be difficult—if not impossible—for all requirements to be stated. It is better to work at a higher level and to explain purposes in terms of goals, and, in certain circumstances, to have the system determine the appropriate path of action. This approach can aid system design and clarity, which leads to the next attribute. The final attribute is a little bit different and perhaps less obvious; namely, the software should be understandable, at least in its design and overall purpose. We need ways of thinking about and describing software that simplify complexity, at least with regard to describing behavior. The desire for understandability is influenced by the software engineering perspective undertaken within this book. There are many potential advantages of understandable software, including better instructions for how to use it.”

Hence, once the system components and behavior relationships are understood even in complex settings, artefacts can be more easily designed, maintained, and developed further. This finding calls for contextual and explicit representations of intelligent behavior (cf. https://www.darpa.mil/program/explainable-artificial-intelligence, https://arxiv.org/abs/2006.00093) (accessed on 4 February 2021). They not only facilitate dynamic arrangements of components and complex systems, as required for CPS development, but also help ensure transparency from a stakeholder perspective.

## 4. Capturing CPS Behavior

We start out with the concept of behavior encapsulations for single system components. We proceed with design-relevant patterns from multiagent environments and detail them in terms of subject-oriented representation.

### 4.1. CPS Components as Self-Contained Subjects

Behavior encapsulations stemming from agent systems are considered (re-)active components, processing inputs from an environment and producing output according to their processing scheme, which cause an environment to change. In particular, they might interact before they react on environmental inputs (depending on the precondition associated with their actions) involving other agents. Trying to identify the best action in the current situation, agents decide which of their actions is performed according to the inputs provided. Finally, agents can act proactively, requesting data from the environment to check whether further interventions are required.

**Figure 7:**
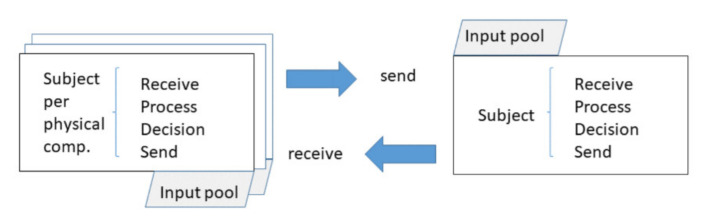
CPS components as subject representations used for digital twin representation—each physical and digital component becomes a subject.

Figure 7 shows how subjects capture CPS components as digital twin based on the agent conceptualization in Figure 2 and Figure 3. Each subject in that environment is able to receive inputs (stored in an input pool). Processing this input allows decision-making before either proceeding with actions and/or sending messages to other subjects. Each physical CPS component, e.g., a sensor system, is represented as a subject. The sensor data are considered as the input (“Receive”), whereas actuators are controlled through actions (“Process”). The fundamental difference between agent-system representations (according to Wooldridge [7]) and subject-oriented ones is that the latter allow representing explicitly all effects an agent’s reaction or action has. In subject-oriented models, agent behavior is encapsulated, regardless of whether activities are set in a reactive, proactive, or social way. When behaving reactively, subjects (CPS components) receive inputs from their environment that trigger appropriate actions in response to external events and changes that occur in the environment. When behaving proactively, subjects take initiative and perform self-contained, goal-directed actions. When acting in a social way, subjects interact via cooperation, coordination, and negotiation with other subjects (and, possibly, with humans) to perform specific tasks according to a common goal.

**Figure 8:**
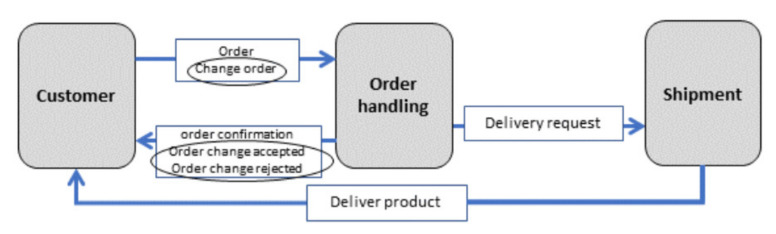
Subject-Interaction Diagram including a change of order.

**Figure 9:**
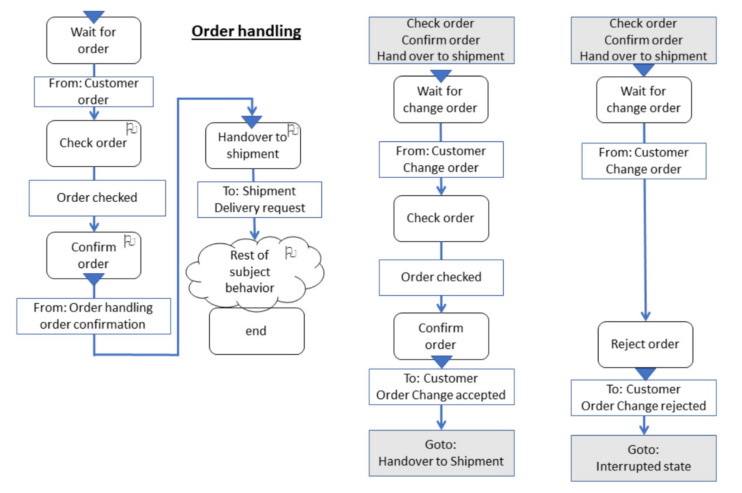
Order Handling Subject Behavior Diagram (SBD) including a change of order.

Figure 8 and Figure 9 show subject-oriented representations of a proactive behavior from the perspective of the Order Handling subject, as the acceptance of the change-order request precedes the delivery request. Both on the level of Subject-Interaction Diagrams (SIDs, Figure 8) and on the level of Subject-Behavior Diagrams (SBDs, e.g., in Figure 9) changes of customer orders can be specified, enriching the behavior of CPS. According to the S-BPM, the subject (CPS component) Order handling “waits” for the respective input (message) to start decision-making on the change of order. In this way, the proactiveness of the concerned organization becomes visible, and behavior patterns can be adapted accordingly.

In the grocery shop use case, a proactive assistant CPS component sends a message to the refrigerator as CPS component to check which items are there. Upon receipt of the available item list, it prepares a shopping list proposal and sends it to the customer. Reactive behavior could also be of benefit—namely, in case the customer has forgotten an item that needs to be added after having triggered the ordering process.

**Figure 10:**
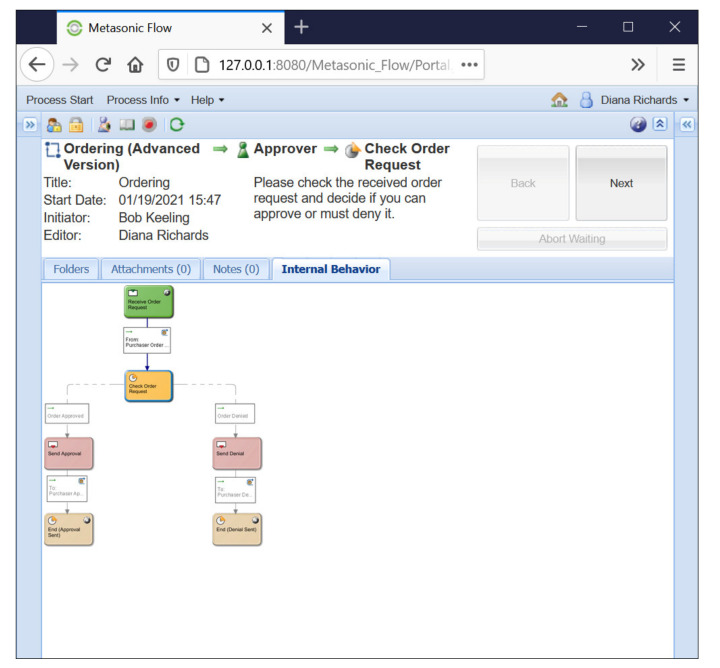
Subject-oriented representation tested interactively (using the Metasonic Suite—metasonic.de).

**Figure 11:**
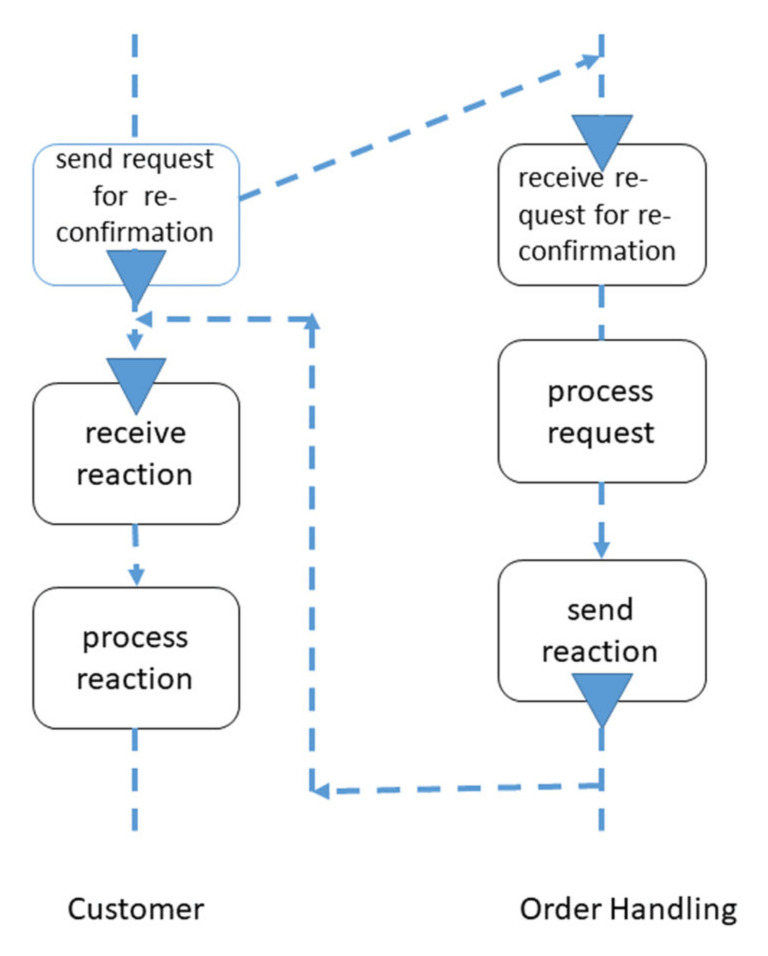
Proactive behavior pattern for a customer request on an order change.

Executing subject-oriented representations allows testing (categories of) the behavior interactively, as shown in Figure 10, for approving order requests. Each subject-oriented model can be instantiated for validation to check the semantic coherence of the represented behavior. Proactive behavior, as the label indicates, is a role behavior requiring some commitment prior to action. Figure 11 displays a proactive pattern in subject-oriented notation that enables commitment for action before an activity is actually set.

Such a pattern-based approach can be a first step towards representing adaptive behavior. Stratulat et al. ([19], p. 813) recognized the challenge of capturing interactions between agents, respecting the autonomy and self-organization of system components: “Agents are considered as active autonomous components with respect to the control of their behavior and to the relationship they have with other components of the system, i.e., the other agents and the environment in which they act. The various studies in the MAS (Multi-Agent System) domain showed that the interaction in an agent-based system has multiple facets that are difficult to grasp. The consequence of this is that the basic concepts and principles of MAS have been identified and studied much of the time in isolation from the others and the various perspectives that are adopted are often orthogonal and mutually exclusive (e.g., internal vs. public meaning of the communication, rational vs. reactive internal architectures, agent-based vs. environment-mediated interaction, agent-oriented vs. organization-centered MAS, etc.). However, it seems that if we want to have a deeper understanding and a more complete perspective of the interaction process some efforts should be made to rethink all the basic elements and to integrate as many as possible of them into a unifying framework.” Therefore, in their paper, they described a general framework and an abstract model “of what constitutes a first step towards an integral view of agent-based interaction” (ibid.).

Sterling et al. ([6], 9f) highlighted several qualities of agent-based components that are also relevant for CPS. Firstly, purposefulness: It denotes pursuing a goal and includes a corresponding sequence of actions to achieve that goal. Secondly, controlled autonomy: Therefore, (CPS) components have the capability to strive for individual goals in an independent way. Finally, components’ situatedness means their awareness of their environment—namely, in terms of perceiving changes in the environment and determining specific actions to respond to them. Subject-oriented models allow representing of each of the addressed qualities:

- Modeling starts out as the relevant goals determine the specifications of the subjects—they encapsulate goal-oriented behavior.
- According to the goal that leads to its identification, a subject encapsulates the behavior that enables to achieve that goal. In this way, purposefulness is implemented.
- Since a subject contains encapsulated behavior, it also is able to pursue a specific goal independently from other subjects—it has controlled autonomy.
- Each subject interfaces its environment namely in terms of sending and receiving messages carrying data to be processed. As such, a subject is not only situated in that environment but also context-aware, since, through exchanging information with another subject, relevant context information is delivered to other components. By sending messages to other subjects, it influences the environment.
- Due to the bidirectional capabilities active and passive behavior with respect to awareness can be represented, either through requesting information about the behavior of other subjects of the environment (active behavior) or through receiving information on changes of the environment and responding on demand (passive behavior).

In particular, the latter property helps to actively collect information of relevance to change behavior—see Section 4.

### 4.2. CPS as Networked Multicomponent Architectures

In a component system modeled as a multi-agent system, a collection of software agents collaborate in an external environment via their sensors and effectors (cf. Figure 4). In that context, Nealon et al. ([18], p. 2) referred to the underlying system characteristics—namely, communication: Agents “can communicate with users or other agents. Thus, they can exchange information, engage in complex negotiations, and coordinate their activities to cooperate in the joint resolution of a problem. Agents usually have reasoning, planning and learning capabilities that allow them to display an intelligent behaviour.” However, the abstraction of multi-agent systems has, so far, received little attention [20]. In particular agent-based modeling is still under development [21]. Most approaches serve implementation and architecture purposes (cf. [22,23]) and only few for strategic business development via simulation (e.g., [8])). Hahn et al. [24] recognized the demand for further research: “Various agent-oriented methodologies and metamodels exist to design and develop multiagent systems (MAS) in an abstract manner. Frequently, these frameworks specialise on particular parts of the MAS and only few works have been invested to derive a common standardisation. This limits the impact of agent-related systems in commercial applications.” In their work, they argued for developing “a metamodel for agent systems that abstracts from existing agent-oriented methodologies, programming languages, and platforms and could thus be considered as platform-independent”. Their metamodel allows defining an abstract syntax of domain-specific modeling languages for MAS to provide for code generation out of generated designs. They apply the principles of model-driven development (MDD) and utilize two model transformations to bring the generated models into textual code that can be executed.

Wooldridge [7] considered multi-agent systems not only as systems composed of multiple interacting intelligent components that interact to solve problems that are beyond the individual capabilities or knowledge of each individual but, also, as decentralized systems without global control and asynchronous computation. However, there is some dispute on the level of granularity that needs to be resolved for design. For instance, Cohen et al. [20] investigated the state space of MAS and possibilities to reduce its size. In order to achieve a tractable size, MAS can be represented in an abstract way. The resulting models for MAS capture temporal-epistemic properties in order to support model checking through fitting MAS state spaces.

For the sake of computational checking, local states and actions of agents are simplified, which points into the direction of subject orientation. Checking design requirements as expressions in temporal-epistemic logic in a simplified approach is based on the assumption that the requirement holds for the entire system that is concerned. Consistent with subject-oriented systems, MAS contain local states and a local protocol for accomplishing task and the capability to individually develop each node of a network. Modeling multi-agent systems according to [25], each component is described by the following elements:

- possible local states a component such as a network node (agent) can be in.
- actions a network node is able to perform.
- a local protocol that allows selecting actions—depending on the current local state of a networked component.
- a local evolution function. It captures the logic how a networked component evolves from one local state to another. This step depends on the component’s own action and the actions set by other ones, set out by communication.

This approach is in line with subject orientation. On the one hand, Subject Behavior Diagrams (SBDs) encapsulate all possible local states of networked nodes in terms of a subject—namely, in terms of send, receive, and act. On the other hand, this subject is also part of the Subject Behavior Diagrams (SBDs) that have been designed to denote the behavior encapsulated by each subject. The local states of a (CPS) component represent the actions it is able perform. They also include the interfaces to other subjects. The local protocol is represented in Subject Behavior Diagrams by modeling all actions that can be triggered internally (do) or externally (send).

According to the subject-oriented interaction paradigm, the protocol specifies receiving a message that corresponds to execute an action of the subject represented by the SBD. The messages that are received by subjects can invoke special behavior sequences serving the evolution. Therefore, the behavior proceeds from one local state to another. The trigger for that special step is implemented in the network node’s own actions; however, it can be triggered by the actions of other subjects. The step is set when processing the incoming messages from other network components (subjects).

In the MAS framework developments, initially, the environment is left out (cf. [26]), which is not the case for subject-oriented representations. The explicit embodiment of subjects (agents) in their environment is, rather, seen as a prerequisite for further developing intelligent behavior and, thus, the intelligence of systems, such as organizations.

Proponents of MAS claim that a model of an agent should be “constructed to aid in building the system that we have in mind. To paraphrase Parnas’s well-known characterization of specifications, a model should be as complex as it needs to be to reflect the issues the system is being built to address, but no more complex” ([6], p. 14). Given the example provided by the authors in UML (Unified Modeling Language—see also http://uml.org/) (accessed on 4 February 2021), models should not only be “intuitively understandable” but, also, support the construction of software systems at a level that allows model checking and reviewing in terms of system correctness before the code is generated and the system is implemented. Consequently, interfaces between components, such as classes, subjects, or agents, need to be defined. Additionally, the passing mechanisms of information need to be specified while “the models must have sufficient detail to be useful, but not so much detail as to overwhelm” (ibid., p. 15). North et al. [8] went one step further, considering modeling as an essential prerequisite for simulation. An MAS model therefore has to contain:

- A set of agents (part of the user-defined model)
- A set of agent relationships (part of the user-defined model)
- A framework for simulating agent behaviors and interactions

For simulations, unlike other modeling approaches, modeling is driven by the perspective of autonomous decision-making units. Therefore, an agent representation, such as subject-oriented models, needs to capture the rules of behavior operating affecting the attributes. The decision rules vary for every intelligent component (subject) in terms of sophistication and representations of the external world. Simulation is based on “local” interactions among the components, since no central authority or controller exists for how the system is modeled, how it operates, and how the system/model moves from one state to another. These objectives can be met by MAS as networked systems represented by subjects—in particular, due to their choreographic arrangement and execution capabilities:

- Each subject has its individual intelligent behavior, represented by state transitions triggered by inputs and dedicated decision rules.
- The interaction among subjects is not disturbed by any supervising authority. It is driven by individual subjects sending and receiving messages. Hence, the overall CPS corresponds to choreographed self-contained units (subjects).

Consequently, any optimization of a system composed by subjects is based on the intertwining of individual behaviors (subjects) through communication. A rule-based approach for governance has been introduced by North et al. [8], following theories of rational behavior of individuals. These assume individuals, in order to survive, collaborate with others when it is in their best economic interest to do so. The authors also suggest simple rules, as (i) they facilitate decision making simple rules and (ii) allow complex rule structures to evolve. These mechanisms are considered helpful to model bounded rationality and adjacent reasoning procedures. The latter are utilized to successively refine rule sets, as the currently available knowledge influencing the abilities of individual decision-makers requires continuous revision and development.

**Figure 12:**
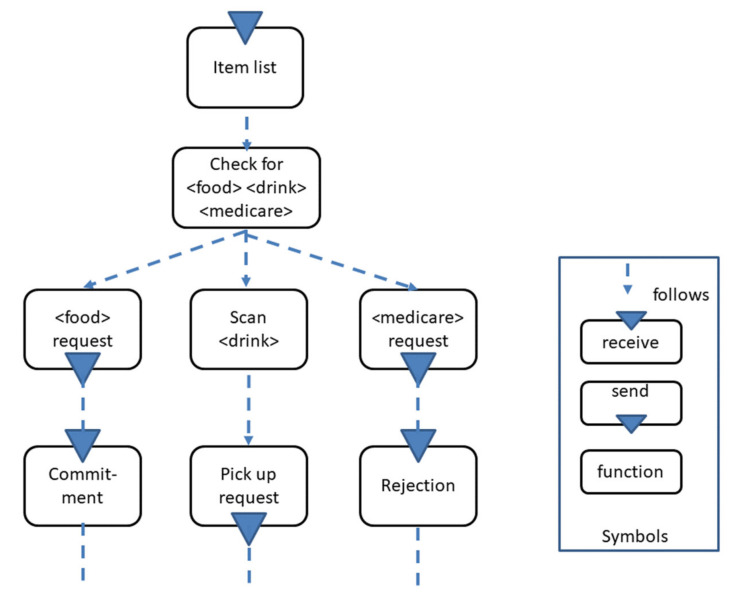
Exemplified decision-making behavior pattern.

In line with that, I propose to use modular decision-making patterns, as shown in Figure 12. The <terms> represent situation parameters that trigger a behavior, such as accepting, rejecting, or requesting refinement of an order, which, in turn, trigger different behaviors, expressed in the subsequent branches. In the exemplified case, categories of order items are part of the pattern. In the case of <food>, a corresponding send request is activated, accepting the order for food to be committed by a subject picking up the food items. Acting is decided for <drink> items through preparing the request for refinement, i.e., picking up the relevant item(s) from the order. Moreover, Send is activated according to the category <medicare>, leading to a rejection, since the grocery shopping assistant does not pick up the medical goods.

Such structures of MAS can be used to map the shopping assistant behavior to models that can be executed automatically. In case already recognized patterns are used for subject-oriented models, the validation before execution (cf. [9]) and, thus, simulation of handling complex situations can be facilitated.

In the grocery shopping case, conditional shopping in the case of a distraction, logistic interferences, diet requirements, or other customer needs could be met by the decision-making pattern. The pattern can handle default cases and variants in terms of general “otherwise” clauses, e.g., calling for human intervention into the ordering process. It can be standardized to trigger domain-specific behavior once certain situation parameters require it. Moreover, it can be combined with pro- or reactive patterns, establishing more complex behaviors.

**Figure 13:**
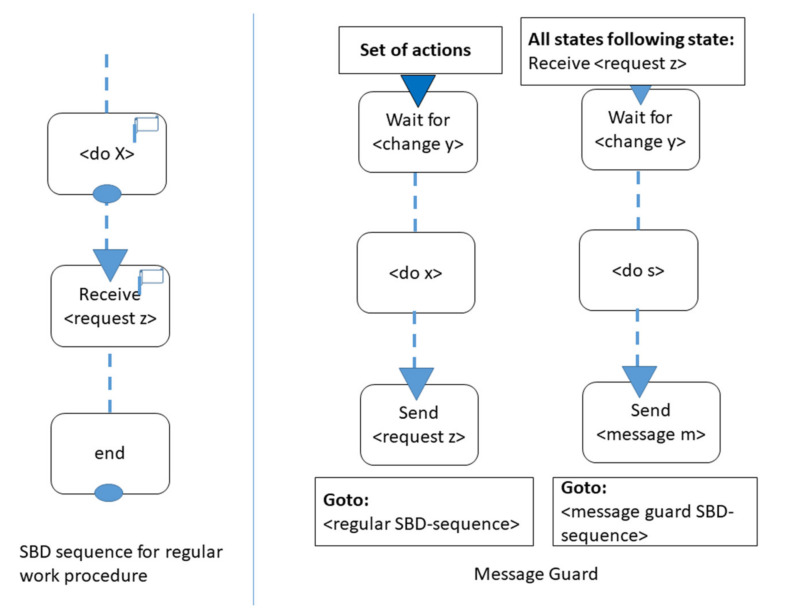
Intelligent behavior specifications: modeling how to handle deviations from routine behaviors.

In case of deviation from the default behavior, exception handling can be supported at design time. Throughout modeling, nonstandard behaviors can be distinguished from routine ones; however, in a context-sensitive way. In Figure 13, the enabler, i.e., the message guard pattern, is pictured. It can be applied when a change of orders occurs. As shown in the figure, specifying exceptions or deviations from standard behavior is assigning a hissing flag (as for receiving the change request for an existing order). This request either triggers a substitutive behavior sequence, including the return to the regular behavior sequence, or leads to a complementary behavior. In the latter case, the originally specified behavior is abandoned, and another behavior sequence is modeled without returning to the original behavior sequence.

Once variants of a behavior emerge, each functional state in an SBD that can be affected is “flagged”. It indicates that alternative behavior sequences can be executed upon request. For instance, consider the grocery shopping assistant moving to stock and requiring extra permission for alternative goods in case the requested one is not available. It may receive “skip this order item”, “take it”, “put it on a waiting list”, or other answers. In any case, the flag to change the regular behavior is hissed. The pattern in Figure 13 shows the two general exception handling behavior patterns: either an additional sub-process is executed before the original procedure can be completed or a completely different behavior is specified once the flag is hissed.

## 5. Choreographic Intelligence Deployment

In CPS, due to the heterogeneity of the components and dynamics of an operation, components need to be aware of situations and behavior specification sensitive to changes. In this section, we focus on the subject-oriented conceptualization with respect to adaptive behaviors based on sensed data. For adaptation subjects, there is a need to (i) become informed and (ii) selective with respect to their behavior. The approach was inspired by Gero et al.’s proposed architecture for situated agents [27,28], implementing their architecture by dedicated communication patterns. Hereby, the sensors of a subject are captured through monitoring. As it can bundle several actions, a dedicated subject specification for that purpose is proposed. It contains activities to (actively) search for and recognize relevant changes in the environment. In that proactive approach, each relevant item is considered as direct input for an acting subject and provided to the respective subject.

For decision support, a similar modeling approach can be taken. Gero et al. [29] denoted a respective component hypothesizer. It can be activated by any subject that requires respective support. A decision support subject received with monitored information for processing, as it is considered situation-sensitive data. Decision support can be provided through identifying mismatches between the current and a desired situation. According to Gero et al., decision support for an acting subject through “deciding on actions that when executed are likely to reduce or eliminate that mismatch” ([29], p. 102). Based on the results of the decision support process, the acting subject can decide which sequence of operations to execute.

**Figure 14:**
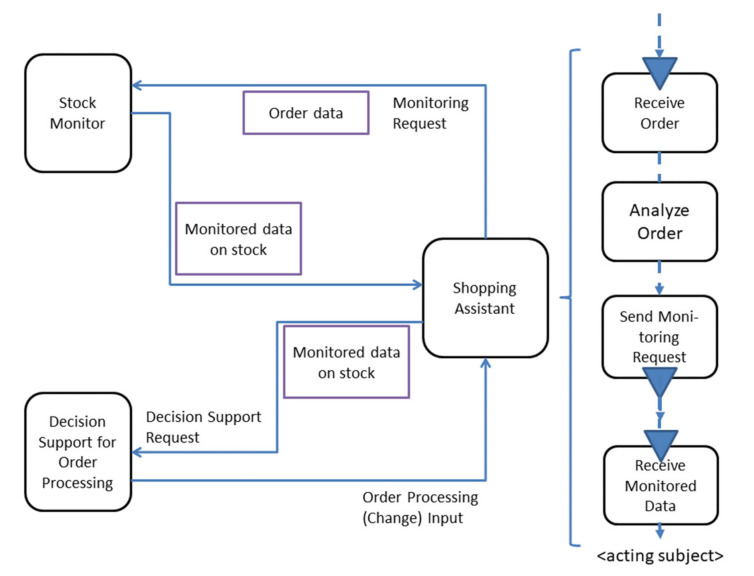
An <acting subject>, here: Shopping Assistant, can ask for monitoring to decide in a situation-sensitive way.

Figure 14 provides the corresponding interaction schema for communication-based engineering of agent-based environments. The figure displays a Subject Interaction Diagram. It reveals the embodiment of an <acting subject>. We denote an <acting subject> as subject that is involved in a decision-making process. On one hand, it can ask for monitoring support given a specific situation, and, on the other hand, according to its connection to a decision support subject, it can process decision-relevant information, even based on the information delivered by the monitoring subject. Hence, systemic intelligence requires three types of components:

- <acting subject>: This type of component is a subject is acting to accomplish a specific decision-making task, e.g., to complete order handling.
- Monitoring: This type of component is capable to receive and process requests to collect data on the current situation through its message passing connections. It is also able to automatically and push information based on received and processed data from its environment to support the <acting subject>.
- Decision Support: It supports selecting the next action and is consulted by an <acting subject> in a certain state. It is provided with all available information on a certain situation through this subject, which is coupled to monitoring.

Figure 14 shows the pattern applied for the shopping assistant. For better understanding, a relevant part of the behavior of the <acting subject>, i.e., the Shopping Assistant, is detailed on the right side of the figure. It shows the receipt of a shopping order and its subsequent analysis. In order to ensure complete delivery, the <acting subject> sends a request to the stock monitor, including the order. Upon receipt, it checks whether the order can be completed. In the case of expecting an incomplete order, handling a decision support subject for order processing is activated and provided with the monitored stock data. The received data concern the input on changing the order, which might have to be reported to the customer.

This pattern enables to apply specific decision-making frameworks established in business operations and, also, avoids decision-making bias (cf. https://medium.com/swlh/3-frameworks-for-making-complex-decisions-6a77099c9683) (accessed on 4 February 2021). The pattern relating an <acting subject> to monitoring and decision-making couples contextual data to a specific business case and, thus, resolves practical handling problems of decision support—in particular, concerning the validity and effectiveness of the decision against the actual course of events (cf. https://hbr.org/1967/01/the-effective-decision) (accessed on 4 February 2021). The actual course of events is captured through the monitoring behavior encapsulation and feeds into the decision support component through the timely flow of information under the control of the <acting subject>, including the respective action to be set when implementing a decision, e.g., reporting to the customer.

**Figure 15:**
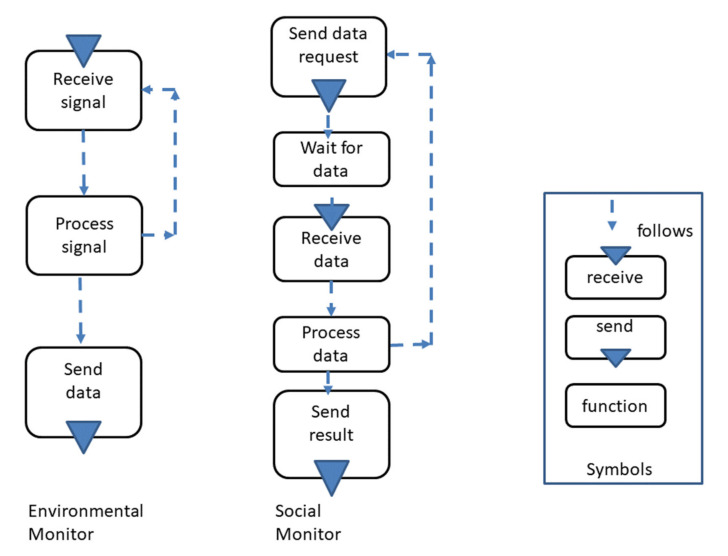
Monitor variants, environmental monitoring as a functional delivery of data, and social monitoring.

The control flow of monitoring can either be environmental or social. As shown in Figure 15, monitoring can be environmental monitoring (left side) referring to monitoring behavior as signal recipient and processor in the course of data delivery. For instance, the room temperature is measured by a respective sensor that recognizes the value as a signal (“receive signal”) and interprets it as a temperature (“Process signal”) before the information is delivered to an acting subject. Once a sensor or <acting subject> automatically sends data to another subject, a monitoring subject can receive and deliver environment data automatically to another <acting subject>.

In the case of the grocery shopping assistance, many different application cases can be included, e.g., conditions for frozen food need to be monitored. In case the temperature is too low, specific alarm procedures need to be activated: For instance, logistics for frozen goods need to be checked in terms of distance and alternative cooling facilities of the carrier; in addition, the producer of the frozen goods needs to be informed of delivery problems.

In addition to environmental monitoring, Figure 15 shows the second type of monitoring—namely, social monitoring. In that case, the monitoring subject actively requests data from other subjects. This type can be used to poll at certain times whether changes happened in the environment or which value at a certain that time is given. An example for the latter is measuring the temperature at 12 o’clock every day during the summer in order to determine the mean temperature in that period of the year. The autonomy of the monitoring subject depends on the processing part and, thus, how the communication with the <acting subject> is organized.

When an <acting subject> is involved in social monitoring, it needs to send a request and wait for the result. Therefore, monitoring information, such as a calculated value of the monitoring subject, is further processed by the <acting subject>. The monitoring of any type can be included in a model prior to an action (i.e., function state) of the <acting subject>. In this way, critical steps in processes can be secured through providing monitoring information. Depending on the result of that, preprocessing the subsequent action can be adjusted. Such a capability is required for sensitive CPS applications, e.g., when environmental data beyond subject interactions are collected and require corresponding action.

**Figure 16:**
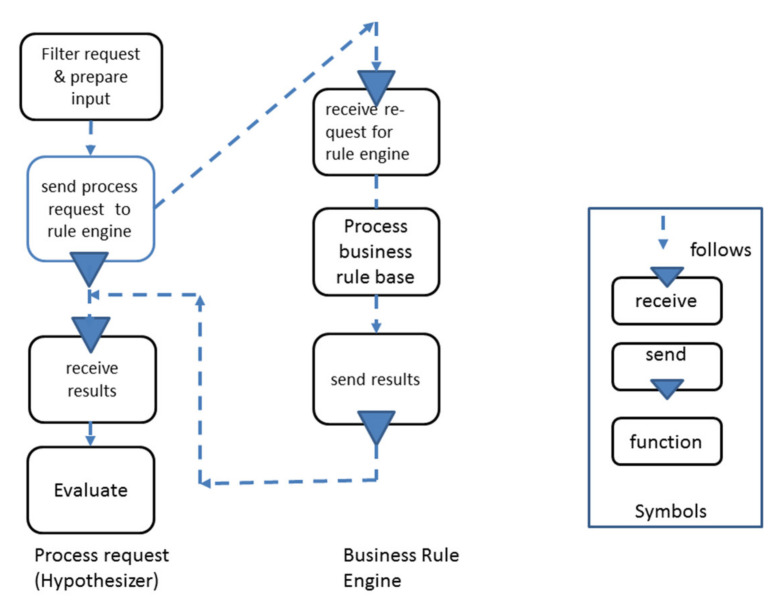
Integration of rule processing.

Once an <acting subject> receives monitor data, it can act in response to environment data. If the input data from Monitor require further checks for operational reasons, a decision support routine needs to be activated. As shown, a Decision Support subject is invoked and is provided with initial information. It could start requesting more data for inference information concerning a specific situation. In some cases, e.g., ensuring food storage, regulations and standards play a crucial role. These can be provided for CPS as rule bases, with subjects governing business- or sector-specific regulations, standards, or conventions. Due to the simple interaction protocol, the relevant input data can be delivered in both directions. As Figure 16 exemplifies, an <acting subject> can be supported in a situation-sensitive way through a rule base.

Rules are activated from actions (i.e., function states), being part of the internal behavior of a subject. They provide extra business or technological logic to run CPS operation. Depending on the result of the rule processing, a decision can be made. A Business Rule Engine, as shown in Figure 16, is an extra component but works like any other subject on the messages exchanged between subjects. It is defined through an internal behavior of processing IF-THEN-ELSE constructs in incoming messages. These constructs can be handled applying the decision-making pattern and checking incoming conditions for the selection of actions.

In a setting where regulations determine interactions among the acting components, e.g., heterogeneous CPS elements, decision support in a modular way through subjects facilitates cross-checks according to different perspectives, ranging from quality standards to legislation. These checks are likely to be performed on technical or functional deliverables in order to complete work tasks. They can be automatically activated, e.g., in case a certain threshold is given. For instance, the shopper assistant subject checks its order list and needs to decide whether to buy an alternative product, e.g., in conformance with a person’s diet plan, while being active in shopping, since the originally intended one is not available (cf. Figure 3).

For that reason, the shopping assistant subject triggers monitoring, as shown above, but, this time, to help identify other products in stock that fit to the same category of goods. In the case of success, the alternative product is checked for fitting the customer’s purpose. The Decision Support subject may require additional consumer behavior (data) to check the fitness. Eventually, the customer can be satisfied with an alternative product, or he/she needs to be informed about the situation and further options. When combined with checking the overall satisfaction level of the customer with the current shopping process so far, situation-sensitive CPS behavior can be modeled. Since a situation can be monitored from different perspectives, multidimensional decision-making can be designed in a structured and transparent way. It influences the upcoming actions of the <acting subject> through contextual information.

**Figure 17:**
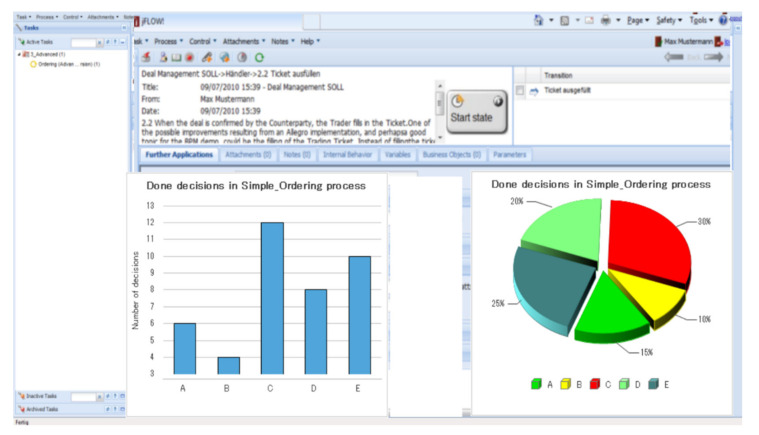
Business Analytics example for simple ordering (using Metasonic.de).

Analytical tool support for the digital twin can be provided for subject-oriented representations, as shown in Figure 17. For instance, for simple ordering, processes can be analyzed according to situational requests, order data, or items that needed to be double-checked, since the original shopping request could not be met. Since the subject execution couples runtime behavior to the digital twin model, decision-making and functional activities can directly be located by each stakeholder and become available for design changes.

## 6. Discussion and Conclusions

Cyber-Physical Systems (CPS) are currently evolving as common types of socio-technical systems, intertwining heterogeneous digital and physical components. CPS development started in the context of production transforming industrial systems (cf. [30]) before propagating to service industries and business process management, e.g., [31]. In CPS application development, intelligent behavior is a core target (cf. [32]). Digital transformation towards CPS touches technical and organizational issues, including design and control models—so-called digital twins [33]. In order to speed up the effectiveness of CPS development, this paper revisited the structure of digital twins from an agent-based and, thus, behavior-centered perspective. Although agent systems have a long tradition in implementing CPS, their concepts have not been utilized for designs that can be executed automatically.

This conceptualization lays the ground for human-centered CPS in intelligent manufacturing systems, going beyond digital and digital-networked manufacturing (cf. [34]). It enables seamless processing cycles of operations like design, production, product, and service. Embodying intelligence is enabled by encapsulating behavior and message passing between CPS components. This modular structure can be used to optimize and integrate corresponding systems. However, human-centered CPS development requires knowledge bases. The represented knowledge addresses machine-to-machine communication (syntax), the meaning of content (semantics), and the preferences of the individuals involved (pragmatics) [35].

The methodological cornerstones for digital specifications mirroring CPS meet the representational demands on the specification level: agent-based systems and subject-oriented modeling. The latter has been explored recently for IoT system specification [36] and task-based CPS design [37], whereas agent-based behavior encapsulation features patterns that have been successfully used to build up system intelligence (given their inherent perception—decision—action sequence). Due to its modular design and easy-to-share notation, subject-oriented modeling allows enriching these patterns towards complex system design while keeping it understandable from a human perspective. Moreover, the models can be executed automatically, allowing for runtime adaptation.

As a result, CPS can be designed in a behavior-centered way to support accomplishing tasks in dynamical and complex socio-technical systems. In this paper, I revisited their structure and behavior from an agent-based perspective, revealing established patterns of design and operation. The suggested subject-oriented models as digital twin representations for CPS enable comprehensive, effective, and efficient system modeling and implementation. The modeling approach takes into account the usability and intelligibility guidelines from empirical studies (cf. [38]) and industrial practices (see, e.g., https://www.heflo.com/blog/bpm/human-centric-bpm/, https://www.heflo.com/blog/bpm/business-process-design-principles/) (accessed on 4 February 2021). The identified patterns use a simple diagrammatic while semantically precise subset of modeling elements for behavior-centered [9] Digital Twin development. They comprise

- active and reactive activities.
- nonroutine behaviors based on events.
- structured integration of business rules.
- decision-making patterns.
- monitoring for informed decision support.

These constructs enable meeting the fundamental qualities of modular intelligent systems—namely, behavior abstraction for a specific purpose and controlled autonomy. In principle, BPMN (Business Process Model and Notation—see https://www.bpmn.org/) (accessed on 4 February 2021) could be used for specifying digital twins, but subject-oriented modeling and validation [9] deliver executable twins while overcoming the limitations of BPMN with respect to choreography (cf. [39]). Besides the dynamic allocation of physical and digital runtime elements to a CPS specification, the federated nature of the system meets the requirements for handling the heterogeneity CPS components through behavior abstraction and for situation-specific adaptation. Therefore, the scope of a CPS is determined by the set of components considered relevant by the concerned stakeholders and the enabling systems supporting the tasks and process accomplishment.

The scope of a CPS is determined by the selected set of interrelated encapsulated behaviors and enables scaling in terms of evolving design elements and technological capabilities used for implementation. Design-integrated engineering is an agile development process, as each adaptation occurs on the digital twin as a CPS-enabled application. The reference architecture of a developed CPS is the currently created subject-oriented model. Thereby, service-oriented architectures can serve as an implementation platform to run the subject-oriented twin and monitor the real-time behavior of the CPS (cf. [40]). Such architectures allow the enrichment of model-based simulations, such as [41], as the models are operationalized for execution.

Future works will focus on processing elicited semantic behavior patterns in several CPS field studies [42,43]. Based on the gained knowledge from the grocery shopping support use case, it will be tested whether largely diverse stakeholder groups will be able to set up behavioral digital twins and dynamically cocreate CPS from a service and production perspective. It is anticipated that collaborate modeling and probing highly interactive and tangible tools, such as Metasonic Touch ^®^ metasonic.de/touch), could be of use to overcome domain-specific and technical barriers—a prerequisite for successful CPS implementation as a socio-technical system. Evaluations will take into account the latest results from transformation studies, e.g., [44], as the introduction of digital technologies in work systems are creating various socio-technical challenges. They do not only affect the performance but, also, the well-being of concerned stakeholders.

## Data Availability

Not applicable.
